# Physiological Distinctions Between Elite and Non-Elite Fencers: A Comparative Analysis of Endurance, Explosive Power, and Lean Mass Using Sport-Specific Assessments

**DOI:** 10.3390/life15101622

**Published:** 2025-10-17

**Authors:** Bartosz Hekiert, Adam Prokopczyk, Jamie O’Driscoll, Przemysław Guzik

**Affiliations:** 1 Department of Physical Education and Sport, Adam Mickiewicz University, 61-712 Poznan, Poland; 2 Department of Sports and Defense Education, Poznan University of Physical Education, 61-871 Poznan, Poland; prokopczyk@awf.poznan.pl; 3 Diabetes Research Centre, College of Life Sciences, University of Leicester, Leicester LE5 4PW, UK; jod16@leicester.ac.uk; 4 Department of Cardiology, St. George’s University Hospitals NHS Foundation Trust, Blackshaw Road, Tooting, London SW17 0QT, UK; 5 Department of Cardiology—Intensive Therapy, Poznan University of Medical Sciences, 61-701 Poznan, Poland; pguzik@ptkardio.pl; 6 University Centre for Sports and Medical Studies, Poznan University of Medical Sciences, 61-701 Poznan, Poland

**Keywords:** fencing, FET, ranking position, countermovement jump, lean mass, heart rate

## Abstract

Fencing demands a unique blend of endurance, explosive power, and asymmetric neuromuscular control. This study compared physiological profiles of elite (top 25 nationally ranked, *n* = 16) and non-elite (positions 26–102, *n* = 33) Polish male fencers using the Fencing Endurance Test (FET), countermovement jump (CMJ), 5-m sprint, body composition, and heart rate (HR) metrics. FET duration, CMJ-derived explosive power (flight time, reactive strength index), and relative lean mass were also assessed in relation to competitive experience. Quantile regression (age & BMI-adjusted), ROC analysis, and Spearman correlations evaluated group differences. Elite fencers demonstrated superior FET duration (median difference: +1.84 min, *p* < 0.0001), CMJ performance (e.g., 10.4 W/kg higher peak power, *p* = 0.014), and relative lean mass (+7.7%, *p* < 0.001), despite comparable 5-m sprint times. Elite athletes also showed more efficient HR recovery (HRR1) and lower pre-FET resting HR (*p* < 0.05). Competitive experience correlated strongly with FET endurance (rho = 0.62), CMJ power (rho = 0.42), and lean mass (rho = 0.55). ROC analysis identified FET ≥ 14.3 min, CMJ flight time ≥0.581 s, and ≥10 years of experience as optimal discriminators of elite status (AUCs 0.86–0.90). These findings confirm that elite performance is characterized by superior sport-specific endurance and explosive power, independent of age/BMI. The FET and CMJ emerge as practical tools for monitoring training progress, with identified thresholds serving as benchmarks for elite preparation. Training programs should prioritize individualized development of these traits, acknowledging inter-athlete variability in physiological strengths. Future research should explore sport-specific acceleration metrics and extended FET protocols for elite athletes.

## 1. Introduction

Fencing is a unique Olympic combat sport characterized by rapid, high-stakes exchanges that demand an exceptional combination of technical skill, tactical decision-making, and physical conditioning [1]. It differs from many sports due to its asymmetric movements, where one side of the body (like the weapon arm) dominates, and its intermittent activity profile, making its physical demands unique compared to continuous, symmetrical sports [2]. While it shares traits with combat sports like boxing, karate, and wrestling (such as explosive bursts, tactical duels, and footwork-based distance control), fencing is distinct in its weapon-based biomechanics, relying on linear lunges and extreme asymmetry rather than rotational strikes or grappling. Its ultra-short, high-intensity bouts also create a specialized energy demand, blending strategic similarities with unarmed combat sports while remaining mechanically unique [3,4].

Athletes practicing amateur fencing may practice irregularly 1–2 times a week, while professional competitors must adhere to specific training cycles that involve regular, even daily training depending on the training period [5]. At the elite level, success hinges not only on experience and mental resilience [6] but also on the athlete’s ability to repeatedly produce high-intensity movements while maintaining precision and speed over extended periods of competition [7]. These demands create a complex physiological profile, blending elements of anaerobic power, aerobic endurance, neuromuscular coordination, and muscle resilience.

Athletes engage in short, explosive bursts such as lunges and fleches—predominantly anaerobic actions—interspersed with prolonged footwork sequences that require sustained aerobic energy supply [8]. This stop-start pattern is metabolically similar to high-intensity interval training (HIIT), pushing both anaerobic and aerobic systems to their limits [9]. Over the course of a tournament, fencers cover distances of approximately 1000 m, where the average length of action is 5 s in foil and 15 s in épée, and the ratio of action to break is 1:3 in foil and 1:1 in épée [10]. Research has shown [11] that fencers primarily work in the aerobic zone during competitions and HR remains below the anaerobic threshold or is moderately activated. This stop-start pattern is metabolically similar to high-intensity interval training (HIIT), pushing both anaerobic and aerobic systems to their limits [9]. During a match, fencers engage in repeated lateral and forward motions—often on one side of the body—leading to asymmetric muscular demands [12,13].

To meet these challenges, elite fencers must optimize physiological traits such as muscle endurance, cardiovascular efficiency, and neuromuscular performance with high levels of explosiveness. In particular, the lower limbs play a crucial role in the stretch–shortening cycle, enabling rapid directional changes and powerful lunges [14].

Efficiency and explosive leg power, such as peak power and flight time, have been shown to correlate with fencing success [15]. Additionally, greater muscle mass in the lower body is associated with improved force production and fatigue resistance [16], although this relationship remains understudied within fencing compared to other combat sports such as judo [17]. Aerobic and anaerobic capacity, alongside heart rate (HR) responses, could be significant indicators of a fencer’s preparedness [18]. In particular, post-exercise HR recovery at one minute (HRR1) is increasingly recognized as a marker of cardiovascular fitness [19], yet remains insufficiently explored in fencing contexts.

Research Objectives

No prior study has comprehensively compared the endurance capacity, explosive power, and body composition of elite and non-elite fencers. The aim of this study was to compare elite and non-elite fencers for:

- endurance capacity, and heart rate responses (including HRR1);
- explosive power;
- explosive acceleration;
- relative lean mass as a part of body composition analysis.

This study further examines the relationship between fencers’ competitive experience (years in elite competition) and their physiological profiles (endurance capacity, explosive power, and body composition).

## 2. Materials and Methods

### 2.1. Participants

We studied 49 male competitors, ranging from junior to senior age groups, all of whom were classified in the unified national ranking system of the Polish Fencing Association. Inclusion criteria for the study were as follows:

1. Active member of the Polish National Fencing Team.
2. Medical clearance for high-intensity exercise.
3. Provision of informed consent.

Exclusion criteria included:

4. Any acute injury or illness that would prevent maximal physical effort.
5. Use of medication that could affect cardiovascular response or performance.
6. Failure to provide informed consent.

The study was conducted during the team’s training camp in the summer preparation period of 2022. Their ranking position was between the 1st and 102nd. Athletes were divided into elite fencers, i.e., those who were ranked between the 1st and the 25th position, and non-elite fencers who were ranked below the 25th position in the senior ranking of the Polish Fencing Association. Elite fencers were operationally defined as those ranked in the top 25 positions nationally (*n* = 16, 33% of the sample), representing a natural cutoff that separated the highest-performing tier of athletes in our cohort. While this threshold was not based on formal selection criteria, it created balanced comparison groups (1:2 ratio) for analyzing physiological differences between competitive levels.

### 2.2. Ethical Issues

In this study, the authors collected data from routine and regularly performed tests of fencers called up for the training camps of the National Team of Poland. All the athletes (or their parent/legal guardian) who were tested gave their informed consent to participate in the research. Ethical approval was obtained from the Bioethical Committee at the Medical University of Poznań, Poland (8 June 2022).

### 2.3. Study Design

Endurance was measured using the proprietary FET, a modification of the original test by Weichenberger et al. [20], which is a type of the Multi-Stage Fitness Test.

**Figure 1:**
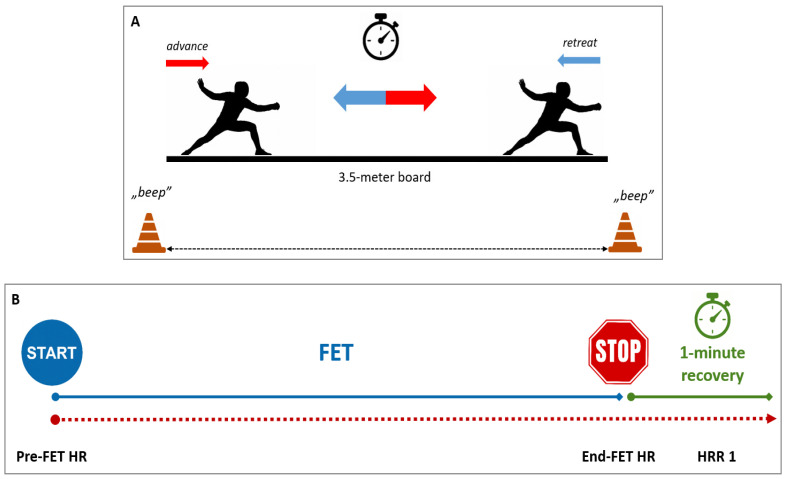
Schematic of the Fencing Endurance Test (FET) (Panel (**A**)) and measurement of heart rate (Panel (**B**)). Panel (**A**). Fencers perform repetitive advance-retreat movements in en garde position, synchronized with accelerating auditory beeps (protocol adapted from Yo-Yo/BEEP tests). Each advance or retreat movement is initiated after a beep sound. The test is continued until volitional exhaustion or 15 min (max). Panel (**B**). Heart rate (HR) is continuously measured by the chest-strap HR monitor. The following values were taken for further analysis: (1) post-warm-up immediately before FET (pre-FET HR), (2) at test termination (End-FET HR), and (3) at 1-min post-test recovery (HRR1).

The FET is conducted on a 3.5 m-long fencing board and is designed to replicate common fencing footwork patterns, including advances, retreats, and lunges. It assesses aerobic endurance in a fencing-specific context by progressively increasing exercise intensity. The test begins at a speed of 3 km/h, with increments of 1 km/h every three minutes. This continues until the participant reaches volitional exhaustion or a maximum duration of 15 min. An audio cue system regulates pacing and ensures uniform intensity progression. FET results are recorded as the total time completed before discontinuation or reaching the 15 min limit. FET is a sport-specific adaptation of the Multi-Stage Fitness Test, based on a modified version of the Beep (or Yo-Yo) Test to assess the aerobic capacity of fencers. Figure 1 illustrates FET and accompanying HR measurements.

The same test applies to junior and senior fencers, regardless of sex [21]. Due to the participation of all the tested competitors in the senior category competitions, the ranking position was determined based on the current position in the senior ranking of the Polish Fencing Association.

The competitors’ height, mass, and body composition were measured in the morning on an empty stomach, before breakfast, at 7:30 a.m. [22]. Participants were not taking any dietary supplements. Nutrition was provided by the Central Sports Center, where the training camp was held. All participants were in preparatory training period. Body composition analysis was conducted using a segmented body composition analyzer, TANITA MC-580 S MA, which operates on the bioelectric impedance method. This method involves passing a low-intensity current (25 VA) of different frequencies (6.25 kHz, 50 kHz) through soft tissues and measuring their electrical resistance. It is suitable for adolescents and adults and is characterized by fast and accurate measurements (error < 1%). For the analysis, we used relative lean mass, defined as lean mass (in kg) normalized to total body weight. While bioelectrical impedance devices often label this parameter as “muscle mass,” it more accurately represents lean body mass, which includes not only skeletal muscle but also organs, skin, and body water. However, skeletal muscle constitutes the largest component of lean mass, and changes in lean mass are primarily attributable to variations in skeletal muscle mass. For this reason, relative lean mass was used as a practical proxy for skeletal muscle mass in our analysis.

HR was measured continuously using a transmitter placed in a chest strap (Polar Team Pro, Polar, Finland) before the FET, after 10 min of warming-up, and during the FET and in post-test recovery. For the analysis, we selected HR:

- before the FET (Pre-FET HR),
- at the end of FET (End-FET HR),
- acquired 1 min after the FET was terminated (HRR 1).

#### 2.3.1. Countermovement Jump (CMJ) Protocol

**Figure 2:**
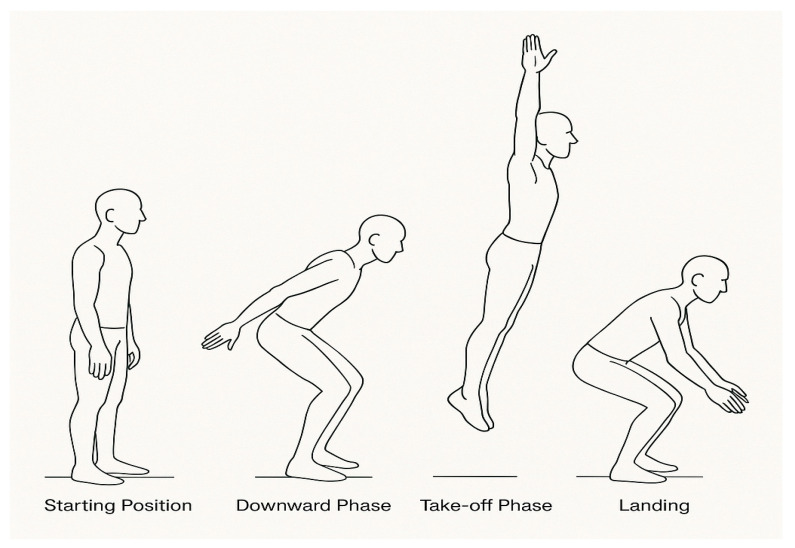
The CMJ test consisted of four consecutive counter-movement jumps (4 × CMJ) with an arm swing.

The CMJ test consisted of four consecutive vertical jumps with natural arm swing (Figure 2). Participants began the CMJ test from an upright position, with feet parallel and hip-width apart. Each jump was preceded by a downward movement (the countermovement) followed by a dynamic, maximal intensity upward propulsion. After each landing, the participant immediately transitioned into the next jump without pausing.

Measurements were conducted using the OptoGait measurement system (Microgate, Bolzano, Italy) [23]. This system recorded the following parameters:

C_time [s]: contact time—the duration during which the fencer is in contact with the ground before takeoff (i.e., the time from the start of the CMJ until the feet leave the ground).

F_time [s]: flight time—the duration during which the athlete is airborne (from the instant the feet leave the ground until they touch down again).

Power [W/kg]: calculated using the formula: Power = (g^2^⋅F_time⋅(F_time + C_time))/(4⋅C_time), where g is gravity acceleration. For analysis, we used the peak power output (highest value) from four consecutive CMJs. Additionally, we measured the mean power of these four CMJs (Power of 4 CMJs),

Rhythm [jump/s: Rhythm expressed in a number of jumps per second.

RSI [m/s]: Reactive Strength Index, calculated using the formula RSI = (g⋅F_time^2^)/(8⋅C_time), to assess a fencer’s explosive power and efficiency in utilizing the stretch-shortening cycle during a CMJ.

The CMJ is particularly important for examining elite versus non-elite fencers as it effectively assesses an athlete’s explosive power and reactive strength, both critical for the rapid changes in direction and explosive movements inherent in fencing. Analyzing CMJ performance can reveal key differences in neuromuscular performance with high level of explosiveness and lower body power development, offering insights into the physical attributes distinguishing high-level fencers from their less experienced counterparts.

#### 2.3.2. 5-Meter Sprint Test

The 5-meter (5-m) sprint test assessed explosive acceleration that is a critical component of fencing performance. Participants began in the en garde position (feet staggered, knees bent, weapon arm ready) on a marked 5-m track, with time recorded using electronic timing gates. Upon an auditory start signal (whistle), fencers sprinted maximally over the 5-m distance. Each participant completed three trials, and the fastest time was retained for analysis to account for intra-individual variability. This protocol simulates the short, explosive bursts required for offensive attacks or defensive retreats during bouts.

All tests were performed during one morning training session with 1-h rest breaks between the tests. The order of testing was as follows: 5-m sprint test → 1-h break → CMJ → 1-h break → FET.

### 2.4. Statistical Analysis

Visual inspection of histograms and Q–Q plots, along with the Shapiro–Wilk test, indicated that most variables did not follow a normal distribution. Consequently, continuous data are reported as medians with the 25th and 75th percentiles (Q1 and Q3, respectively).

Group comparisons for unadjusted continuous variables were conducted using the Mann–Whitney U test. Since age, body height, weight, and the derived body mass index (BMI) differed significantly between the groups, quantile regression was used to compare FET and squat jump descriptors while adjusting for age and BMI. This approach estimates differences in the conditional median between groups, controlling for covariates. Results are presented as regression coefficients (β) with corresponding standard errors (SE), representing the estimated median difference between groups.

In addition to the group comparisons, the association between years of competitive fencing experience and FET results was assessed using Spearman’s rank rho correlation coefficient due to non-normal distribution of variables.

Receiver Operating Characteristic (ROC) curves with respective Areas Under the Curve (AUC) were used to evaluate the discriminative power of FET, HR measured before and during FET, CMJ metrics, and relative lean mass in classifying elite (top 25 national ranking) versus non-elite Polish fencers. Optimal cut-off values were determined using Youden’s index (maximizing [sensitivity + specificity − 1]).

Statistical significance was set at *p* < 0.05 for all analyses. Analyses were performed using PQStat and JMP^®^ Pro 18.0.0 (build 622753) (JMP Statistical Software, Cary, NC, USA).

## 3. Results

The median age of the elite fencers was nearly 21 years, and that of the non-elite fencers was nearly 17 years. The highest ranking was 1st place, while the lowest was 102nd in senior ranking of the Polish Fencing Association. Sixteen participants were placed among the top 25 Polish fencers. These were considered elite fencers in contrast to the remaining 33 non-elite peers.

### 3.1. Comparisons of Elite vs. Non-Elite Male Fencers

Table 1 presents the summary statistics and unadjusted comparisons between the top 25 nationally ranked male Polish fencers and their lower-ranked counterparts (positions 26–102).

Elite fencers were significantly older, taller, and heavier, with greater lean mass and longer competitive experience compared to the non-elite group. They also performed significantly better in FET, showing longer duration and superior recovery. Notably, elite fencers had greater CMJ-derived explosive power, demonstrated by longer flight time, greater maximum and mean power, and improved RSI. Relative perceived exertion was lower in the elite group, despite higher physical output, suggesting better efficiency. No significant group differences were observed in a 5-m sprint speed, pace, or maximum heart rate during FET.

After adjusting for age and BMI using quantile regression, significant differences remained in several key performance metrics. Elite fencers had a significantly higher FET duration, flight time, maximum and mean power output, and RSI. These adjusted results confirm that even when controlling for age and body composition, elite fencers exhibit superior endurance and explosive leg performance. Differences in starting HR, RPE, and HRR did not reach statistical significance after adjustment. The estimated median difference (EMD) between elite and non-elite fencers in FET, CMJ, and HRR is presented in Table 2.

### 3.2. Associations of the Competitive Fencing Experience with Other Measures

Nonparametric Spearman correlations between years of competitive fencing experience and FET results, RPE, lean mass, CMJ, and HRR are presented in Table 3. FET duration showed a strong positive correlation with years of competitive experience, suggesting significantly better endurance capacity. CMJ-derived explosive power metrics (F_time and Power of 4 CMJs) exhibited moderate positive correlations, suggesting that experience enhances repeated explosive movements. Pre-FET HR was inversely related to experience, reflecting potentially more efficient cardiovascular adaptation in veteran fencers. Relative lean mass showed the strongest association (rho = 0.55), among anthropometric measures. Neither acceleration (5-m speed) nor reactive strength (RSI) correlated meaningfully with the competitive experience (all *p* > 0.05). End-test physiological measures (End-FET HR, HRR1) showed no significant relationships.

**Figure 3:**
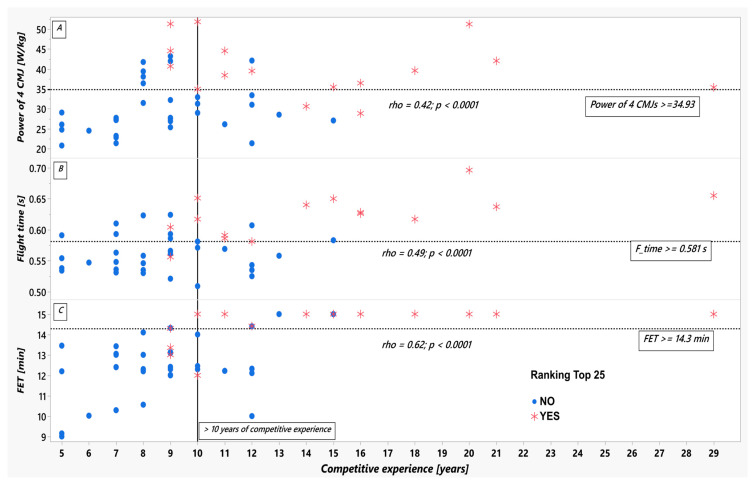
The best three correlations between the years of competitive fencing and features of physical performance results, i.e., the mean power of 4 CMJs (Panel (**A**)), flight time (Panel (**B**)), or FET duration (Panel (**C**)) in a group of elite (red asterisks) and non-elite (blue dots) Polish fencers. The FET cutoff >= 14.3, flying time >= 0. 581 s, and the power of four CMJs optimally separating elite from non-elite fencers, was obtained from the ROC with Youden’s method. Abbreviations: CMJs—counter-movement jumps; FET—Fencing Endurance Test duration in minutes, rho—nonparametric Spearman correlation coefficient; F_time—Flight time.

For the physical performance measures, a medium-strong positive correlation (Figure 3) was found between years of competitive fencing and FET results, the medium with F-time and Power of 4 CMJs. Only pre-FET HR was significantly and negatively correlated with the competitive experience. Among different anthropometric measures, the relative lean mass has a medium-strong correlation with this experience. In other words, more experienced fencers had better FET, F-time, and Power of 4CMJs, a higher lean mass, and a lower HR before FET. As for FET, it is noteworthy that nine out of 16 elite and 2 out of 31 non-elite fencers (*p* < 0.0001 for the comparison of proportions) completed the test and achieved 15 min. All of the fencers who performed FET for 15 min had at least 10 years of competitive experience.

### 3.3. ROC Analysis for Discriminators Between Elite and Non-Elite Fencers

Among the parameters tested for discriminating elite from non-elite fencers, five demonstrated strong classification accuracy (AUC > 0.8) (Table 4). The highest discriminative power was observed for flying time during CMJ (AUC = 0.899), followed by FET duration (AUC = 0.878), power of 4 CMJs (AUC = 0.860), years of competitive experience (AUC = 0.856), and peak single CMJ power (AUC = 0.837). RSI approached this threshold (AUC = 0.793). While pre-FET HR (AUC = 0.678), HRR1 (AUC = 0.685), and RPE (AUC = 0.767) showed weaker but still significant discrimination, relative lean mass, the 5-m sprint speed, and CMJ rhythm failed to differentiate groups (all AUC ≈ 0.5, *p* > 0.05).

## 4. Discussion

This study employed a comprehensive battery of tests —including the FET for endurance capacity, the CMJ for explosive power, the 5-m sprint for acceleration, and body composition analysis—to compare the physiological profiles of elite and non-elite fencers and explore their associations with competitive experience. The results revealed clear distinctions: elite fencers exhibited significantly superior FET performance, greater explosive power as indicated by CMJ (particularly involving trunk and limb muscles), and higher relative lean mass. Elite fencers also had a four-year longer median competitive experience than non-elite fencers. Importantly, these differences in FET and CMJ performance remained robust even after adjusting for age and BMI.

In contrast, both groups demonstrated similar results in the 5-m sprint test, suggesting that raw acceleration—as measured by short-distance sprinting—does not effectively differentiate elite from non-elite fencers. This finding underscores that elite fencing performance is characterized more by sport-specific endurance and power than by general speed. Moreover, the current 15-min FET duration may underestimate the full endurance potential of elite fencers, indicating the need to recalibrate endurance assessments for this population.

Correlation analyses further highlighted that competitive experience was closely linked to enhanced sport-specific physiological characteristics. Fencers with at least 10 years of experience showed longer FET durations (suggesting superior endurance), higher CMJ-derived explosive power metrics (indicative of improved neuromuscular function), greater relative lean mass (implying training-induced muscular adaptation), and lower pre-FET heart rates (potentially reflecting more efficient resting cardiovascular function).

Finally, receiver operating characteristic (ROC) analysis identified FET duration, CMJ-derived explosive power (F_time), and years of competitive experience as the strongest discriminators between elite and non-elite fencers, each yielding an area under the curve (AUC) greater than 0.85. Specifically, thresholds of FET duration ≥ 14.3 min, F_time ≥ 0.581 s, and ≥10 years of competitive experience emerged as critical indicators of elite performance. In contrast, neither body composition metrics nor 5 m sprint times significantly differentiated the groups, reinforcing the notion that fencing excellence is driven more by sport-specific physiological adaptations than by basic anthropometric or speed measures. These cut-off values offer practical benchmarks for talent identification and can inform the design of targeted training programs aimed at developing elite-level fencing performance.

### 4.1. Endurance and Explosive Power: Cornerstones of Elite Fencing Performance

Fencing places significant, integrated demands on both endurance and explosive power. Our findings underscore that these capacities are more critical discriminators of elite performance than isolated speed metrics. The FET and CMJ emerged as highly sensitive indicators, effectively differentiating elite from non-elite fencers. Notably, 56.25% of elite fencers completed the 15 min FET with residual capacity, compared to just 6.06% of non-elites, highlighting the superior ability of elite athletes to sustain high-intensity efforts essential for maintaining tactical precision under fatigue [24,25]. Their enhanced CMJ scores, particularly reflecting greater explosive strength in the trunk and lower limbs, are crucial for rapid directional changes, decisive lunges, and quick recoveries—key offensive and defensive maneuvers. The plyometric nature of the CMJ closely reflects fencing-specific footwork, enhancing reactive agility and force generation [26]. Our data suggest that 14.3 min on the FET serves as a practical threshold for performance stratification, further emphasizing its utility. The lack of differentiation between groups in the 5-m sprint test is particularly noteworthy. This suggests that a baseline level of explosive acceleration might be a shared prerequisite across different skill levels in fencing, or that pure linear sprint speed over a short distance does not capture the multidirectional, reactive acceleration crucial for high-level fencing performance.

### 4.2. The Nuanced Role of Competitive Experience

While the Fencing Endurance Test (FET) and Countermovement Jump (CMJ) scores directly reflect physical preparedness, competitive experience contributes uniquely to a fencer’s overall profile. Competitive experience was found to be a strong discriminator between elite and non-elite fencers, with the elite group demonstrating a four-year longer median experience (13.0 years vs. 9.0 years, *p* < 0.0001). This extensive exposure fosters substantial physiological adaptations; competitive experience exhibited significant positive correlations with FET duration (ρ = 0.62), relative lean mass (ρ = 0.55), and CMJ flight time (ρ = 0.49). Furthermore, pre-FET heart rate was inversely correlated (ρ = −0.50), reflecting potentially more efficient cardiovascular adaptation in more experienced fencers.

However, it is crucial to note that the majority of these significant correlations are categorized as medium to medium-strong (rho values primarily ranging from 0.35 to 0.62). This moderate strength indicates that competitive experience is important, but not the sole or dominant variable explaining the variance in physical performance, endurance, explosiveness, and HR response to exercise. Elite fencers demonstrated strategic pacing and bout management skills are a testament to the synergistic interaction between physical and cognitive attributes. Repeated exposure to high-stakes competition cultivates anticipatory decision-making and psychological resilience [27]. Therefore, while extensive experience provides the necessary context for elite adaptation, superior physical performance must ultimately be attributed to the combination of training quality, genetic predisposition, and other factors, rather than merely the accumulation of training years [28].

### 4.3. Training Implications for Fencing Development

Our findings have potential applications for fencing training and practical control of the preparation of fencing athletes by monitoring the progress of endurance and explosive power. The superior performance of elite fencers in both FET and CMJ suggests that current testing protocols may require refinement. Given that over half of elite participants completed the full 15-min FET with residual capacity, extending the duration to 20 min could provide a more accurate assessment of their endurance limits and better reflect the demands of extended bouts.

In training design, a periodized approach that balances endurance and explosive power is essential [29]. FET-based conditioning can incorporate intermittent, high-intensity bouts (e.g., 3–5 min with equal rest intervals) to mirror the fluctuating effort demands of matches. Simultaneously, plyometric exercises such as depth jumps and weighted CMJs should target reactive strength and rate of force development. The observed 7.7% greater relative lean mass in elite fencers, particularly in the core and lower limbs, supports the inclusion of resistance training (e.g., Bulgarian split squats, Olympic lifts) to optimize power-to-weight ratios [10,30]. These physiological advantages are further leveraged by elite athletes’ superior tactical pacing and bout management, underscoring the importance of a holistic training approach [31]. From a talent development perspective, monitoring relative lean mass (primarily reflecting skeletal muscle) is crucial as it directly supports power output and energy availability during competition [32]. Conversely, the similar 5-m sprint performance across groups suggests that while basic acceleration is necessary, specific training interventions focused solely on short linear sprints may not yield significant advantages in differentiating elite fencing performance.

### 4.4. Future Directions

Several promising research avenues emerge from this work. Validation studies should examine the discriminative power of FET and CMJ in broader populations, including female fencers and international competitors, to confirm the universal applicability of these metrics. Interventional trials could assess whether targeted training programs focusing on specific endurance or explosive power deficits actually improve competitive rankings over time. The integration of advanced monitoring technologies, such as wearable inertial sensors during FET and CMJ testing, might provide more sport-specific movement analysis. Future protocols should also consider implementing extended FET durations (e.g., 20 min thresholds) for elite-level assessment. From a practical standpoint, developing normative data tables for FET and CMJ performance across different age and skill levels would greatly assist coaches in athlete evaluation and talent identification. Further research could explore more ecologically valid measures of fencing-specific acceleration, such as change-of-direction speed or reactive agility tests, to determine if these metrics offer greater discriminative power than the 5 m sprint.

The analysis of the relationship between experience and physical fitness revealed an intriguing sub-group: several sub-elite fencers demonstrated physical test results equivalent to those in the elite category, despite possessing significantly fewer years of competitive experience. This observation highlights two critical points for talent identification. Firstly, these athletes may represent the next wave of elite performers, achieving the necessary physical foundation at an accelerated rate. Their high physical capacity suggests a strong potential to transition to the elite level once they acquire the required tactical maturity, psychological robustness, and accumulated bout experience. Secondly, their current placement in the sub-elite fencers, despite superior physicality, strongly suggests that non-physical factors (such as technical proficiency, strategic decision-making, and psychological resilience under competition stress) are the primary limiting bottlenecks for their final ascent. This finding validates the necessity of future longitudinal studies focused specifically on monitoring the development trajectory of this high-potential sub-group, allowing researchers to determine which non-physical variables are the most crucial predictors of future elite success.

### 4.5. Study Limitations

Several limitations should be considered when interpreting these results. The cross-sectional design prevents establishing causal relationships between the measured parameters and competitive success, necessitating future longitudinal studies. The sample was restricted to 49 Polish male fencers, limiting generalizability to female athletes and other national training systems. The top-25 ranking cutoff (*n* = 16 elite vs. 33 non-elite) was chosen pragmatically to create meaningful comparison groups, though we acknowledge it does not reflect an official selection threshold. Although this approach enabled statistically viable group comparisons and aligned with our observational study design, it may not fully capture the performance continuum in fencing. However, this operational definition successfully discriminated physiologically meaningful differences between performance tiers. The 15 min FET ceiling likely truncated the true endurance capacity measurement for elite performers, suggesting the need for extended test durations in future protocols. Competitive experience was not quantified using objective competition metrics (e.g., international results), which could provide more precise data on performance progression. Additionally, while the findings clearly differentiate elite and non-elite fencers, the relatively small sample size may affect the stability of the statistical models.

## 5. Conclusions

The study revealed clear distinctions in the physiological profiles of elite and non-elite fencers. It was found that superior endurance and greater explosive power are key traits that differentiate elite athletes from less experienced athletes.

The results indicate that a high level of endurance and greater explosive power are directly linked to many years of training experience. The tests used in the study proved to be effective tools for assessing and identifying these key traits.

For coaches, this means that to prepare athletes for top-level competition, they should focus on the development of sport-specific endurance and explosive power. These physical elements, alongside technical skill, are the foundation for success in fencing.

## Figures and Tables

**Table 1:** Comparison of study characteristics between elite and non-elite fencers from the senior ranking of the Polish Fencing Association. These characteristics include results of the fencing special endurance, heart rate, and measurements of explosive power of legs between the top 25 and the remaining fencers (ranking positions 26 to 102) from the senior ranking of the Polish Fencing Association, including results of the fencing special endurance, heart rate, and CMJ-derived explosive power of the legs.

	Non-Elite *n* = 33			Elite *n* = 16			
	Median	Q 25	Q 75	Median	Q 25	Q 75	*p*-Value
Age [years]	17.0	15.6	18.2	21.0	19.2	24.9	<0.0001
BMI [kg/m^2^]	21.4	19.5	24.0	23.5	21.6	24.3	0.0901
Body height [cm]	179.0	174.0	183.0	183.5	180.3	188.0	0.0019
Body weight [kg]	69.0	62.7	77.6	79.6	72.8	82.0	0.0026
FET duration [min]	12.4	12.1	13.3	15.0	14.3	15.0	<0.0001
Competitive experience [years]	9.0	7.0	10.0	13.0	10.0	16.5	<0.0001
Pre-FET HR [bpm]	127.0	114.5	132.5	118.5	110.5	125.8	0.046
End-FET HR [bpm]	198.0	192.0	208.0	196.5	191.8	201.5	0.2625
HRR 1 [min]	161.0	144.0	169.5	149.0	140.5	157.8	0.0386
RPE [point]	9.0	9.0	10.0	8.5	8.0	9.0	0.001
Relative lean mass [%]	59.9	54.9	64.6	67.6	65.2	71.6	0.0004
Duration of the 5-m sprint speed (s)	1.09	1.06	1.13	1.10	1.02	1.16	0.8895
C_time [s]	0.40	0.27	0.53	0.31	0.24	0.44	0.0511
F_time [s]	0.56	0.54	0.58	0.62	0.59	0.65	<0.0001
Power [W/kg]	30.20	27. 59	38.45	42.84	36.40	48.17	0.0002
Mean power of 4 CMJs [W/kg]	27.74	25.71	33.16	39.53	35.39	44.54	<0.0001
Rhythm [jump/s]	1.10	0.92	1.24	1.09	0.94	1.23	0.7087
RSI [m/s]	0.85	0.72	1.28	1.50	1.07	1.73	0.001

Abbreviations: BMI—Body Mass Index; bpm—beats per minute; C_time—Contact time; F_time—Flight time; FET—Fencing Endurance Test; HR—heart rate; HRR—heart rate recovery; RPE—Rate of Perceived Exertion; RSI—Reactive Strength Index; Q—Quantiles.

**Table 2:** Results of the estimated median difference analysis between elite and non-elite fencers in national ranking system.

	EMD	SE	*p*-Value
Fencing Endurance Test [min]	1.84	0.45	<0.0001
Pre-FET HR [bpm]	0.39	6.87	0.9550
HRR 1 [min]	−13.34	8.04	0.0970
RPE—BORG [point]	−0.06	0.33	0.8485
C_time [s]	−0.11	0.08	0.1583
F_time [s]	0.05	0.02	0.0050
Power [W/kg]	10.38	4.23	0.0140
Power of 4 CMJ [W/kg]	10.92	3.99	0.0062

Abbreviations: bpm—beats per minute; C_time—Contact time; F_time—Flight time; FET—Fencing Endurance Test; HR—heart rate; HRR—heart rate recovery; kg—kilogram; min—minutes; RPE—Rate of Perceived Exertion; RSI—Reactive Strength Index; s—seconds; Q—Quantiles; W—Watt.

**Table 3:** Nonparametric Spearman correlation between years of competitive fencing experience and FET results, RPE, lean mass, CMJ-derived explosive power of legs, and HRR of the Polish Fencing National Team.

Years of Competitive Experience	Spearman Rho	*p*-Value
FET duration	0.62	<0.0001
Pre-FET HR	−0.50	0.0003
End-FET HR	−0.26	0.0694
HRR 1	−0.10	0.4833
RPE	−0.39	0.0056
Body height	0.44	0.0015
Body weight	0.48	0.0005
BMI	0.38	0.0067
Relative lean mass	0.55	<0.0001
5-m speed	0.09	0.5239
C_time	−0.17	0.2374
F_time	0.49	0.0003
Power	0.37	0.0081
Mean power of 4 CMJs	0.42	0.0029
Rhythm	0.06	0.6952
RSI	0.35	0.0139

Abbreviations: BMI—Body Mass Index; bpm—beats per minute; C_time—Contact time; F_time—Flight time; FET—Fencing Endurance Test; HR—heart rate; HRR—heart rate recovery; rho—nonparametric Spearman correlation coefficient; RPE—Rate of Perceived Exertion; RSI—Reactive Strength Index; Q—Quantiles.

**Table 4:** Discriminative Power of Physiological and Performance Metrics Between Elite and Non-Elite Fencers: ROC Curve Analysis.

	ROC			Youden’s Index for Cut-Off
	AUC	SE	*p*-Value	
Years of competitive fencing [years]	0.8561	0.0527	<0.0001	≥10
FET duration [min]	0.8778	0.0585	<0.0001	≥14.3
Pre-FET HR [bpm]	0.6780	0.0766	0.0451	≤126
HRR 1 [min]	0.6847	0.0751	0.0376	≤161
RPE [point]	0.7670	0.0734	0.0026	≤8
Relative lean mass [%]	0.5256	0.0867	0.7735	NA
Duration of the 5-m sprint speed (s)	0.5133	0.0963	0.8814	NA
C_time [s]	0.6742	0.0800	0.0498	≤0.472
F_time [s]	0.8987	0.0457	<0.0001	≥0.581
Power [W/kg]	0.8371	0.0558	0.0001	≥32.05
Power of 4 CMJs [W/kg]	0.8598	0.0521	0.0001	≥34.93
Rhythm [jump/s]	0.5341	0.0840	0.7012	NA
RSI [m/s]	0.7926	0.0638	0.0010	≥0.85

Abbreviations: AUC—area under the curve; bpm—beats per minute; CMJs—counter-movement jumps; C_time—Contact time; F_time—Flight time; FET—Fencing Endurance Test; HR—heart rate; HRR 1—heart rate recovery measured 1 min after FET stop; NA—not available; ROC—receiver operator characteristics curve analysis. RPE—Rate of Perceived Exertion; RSI—Reactive Strength Index; SE—standard error.

## Data Availability

The data supporting the findings of this study are available from the corresponding author upon reasonable request.
